# The genetic regulation of the gastric transcriptome is associated with metabolic and obesity-related traits and diseases

**DOI:** 10.1152/physiolgenomics.00120.2023

**Published:** 2024-02-26

**Authors:** Laura L. Koebbe, Timo Hess, Ann-Sophie Giel, Jessica Bigge, Jan Gehlen, Vitalia Schueller, Michael Geppert, Franz Ludwig Dumoulin, Joerg Heller, Michael Schepke, Dominik Plaßmann, Michael Vieth, Marino Venerito, Johannes Schumacher, Carlo Maj

**Affiliations:** ^1^Center for Human Genetics, University of Marburg, Marburg, Germany; ^2^Gastroenterological Centre (Dr. Geppert), Bayreuth, Germany; ^3^Department of Gastroenterology, St. Elisabeth Hospital Bonn, Bonn, Germany; ^4^Marienhaus Hospital Ahrweiler, Ahrweiler, Germany; ^5^Department of Gastroenterology, Helios Hospital Siegburg, Siegburg, Germany; ^6^Gastroenterological Centre (Dr. Plassmann), Bonn, Germany; ^7^Institute for Pathology, Klinikum Bayreuth, University of Erlangen-Nuremberg, Bayreuth, Germany; ^8^Department of Gastroenterology, Hepatology and Infectious Diseases, Otto-von-Guericke University Hospital, Magdeburg, Germany

**Keywords:** expression quantitative trait loci, gene expression, metabolism, stomach

## Abstract

Tissue-specific gene expression and gene regulation lead to a better understanding of tissue-specific physiology and pathophysiology. We analyzed the transcriptome and genetic regulatory profiles of two distinct gastric sites, corpus and antrum, to identify tissue-specific gene expression and its regulation. Gastric corpus and antrum mucosa biopsies were collected during routine gastroscopies from up to 431 healthy individuals. We obtained genotype and transcriptome data and performed transcriptome profiling and expression quantitative trait locus (eQTL) studies. We further used data from genome-wide association studies (GWAS) of various diseases and traits to partition their heritability and to perform transcriptome-wide association studies (TWAS). The transcriptome data from corpus and antral mucosa highlights the heterogeneity of gene expression in the stomach. We identified enriched pathways revealing distinct and common physiological processes in gastric corpus and antrum. Furthermore, we found an enrichment of the single nucleotide polymorphism (SNP)-based heritability of metabolic, obesity-related, and cardiovascular traits and diseases by considering corpus- and antrum-specifically expressed genes. Particularly, we could prioritize gastric-specific candidate genes for multiple metabolic traits, like *NQO1* which is involved in glucose metabolism, *MUC1* which contributes to purine and protein metabolism or *RAB27B* being a regulator of weight and body composition. Our findings show that gastric corpus and antrum vary in their transcriptome and genetic regulatory profiles indicating physiological differences which are mostly related to digestion and epithelial protection. Moreover, our findings demonstrate that the genetic regulation of the gastric transcriptome is linked to biological mechanisms associated with metabolic, obesity-related, and cardiovascular traits and diseases.

**NEW & NOTEWORTHY** We analyzed the transcriptomes and genetic regulatory profiles of gastric corpus and for the first time also of antrum mucosa in 431 healthy individuals. Through tissue-specific gene expression and eQTL analyses, we uncovered unique and common physiological processes across both primary gastric sites. Notably, our findings reveal that stomach-specific eQTLs are enriched in loci associated with metabolic traits and diseases, highlighting the pivotal role of gene expression regulation in gastric physiology and potential pathophysiology.

## Graphical abstract

**Figure:**
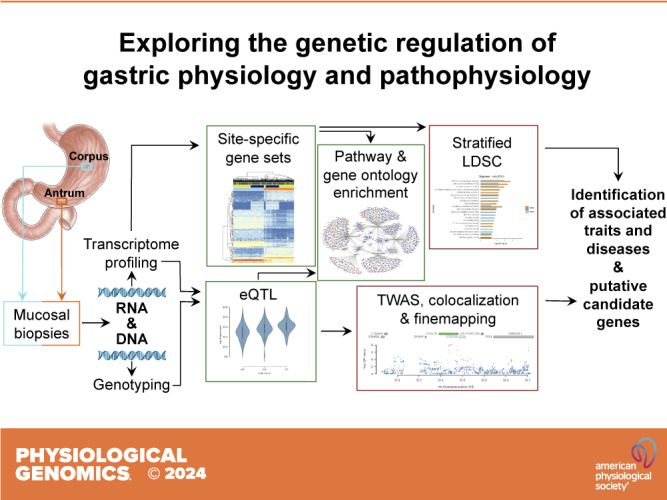

## INTRODUCTION

In contrast to the genome, the human transcriptome does not only vary across individuals but also across tissues and cell types (1). It is well established that genetic variation alters gene expression levels and thereby contributes besides physiological processes also to complex traits and diseases (2). A genetic locus containing a sequence variant that affects the transcript level of a gene is called an expression quantitative trait locus (eQTL) (3). Thus, eQTL mapping is a powerful approach to uncover the genetic component of gene expression variation which is crucial to understand the molecular mechanisms of diseases (4). As eQTLs can operate in a tissue-specific manner and many disease phenotypes manifest themselves only in particular tissues, eQTL analyses are most successful when performed in disease-relevant tissue (5–7). The Genotype-Tissue Expression (GTEx) project has collected eQTL data across 49 human tissues retrieved from up to 838 postmortem donors and provides the largest resource to systematically evaluate the genetic regulation of gene expression (8). The stomach data set from GTEx contains expression data from the corpus which is the biggest of the different gastric regions.

In fact, anatomically, the stomach can be divided into five distinct regions, namely cardia, fundus, corpus, antrum, and pylorus (9). Functionally, it consists of two parts, the proximal and distal stomach. Cardia, fundus, and the proximal corpus build the proximal stomach where the food is stored and most of the chemical digestion takes place (9). The distal stomach, which comprises the remaining part of the corpus as well as antrum and pylorus, is more muscular and mixes the food with the gastric secretions before releasing it to the duodenum (10, 11). The existence of both functional regions can be further explained by different mucosa histology as there are two types of gastric mucosa, the oxyntic and the antral mucosa. Oxyntic mucosa is found in the proximal gastric part and is populated by glands that secrete gastric acid, digestive enzymes, and intrinsic factor (12, 13). Antral mucosa is found in the distal gastric part and contains predominantly pyloric glands secreting mostly mucus to form a barrier that protects the stomach from self-digestion (12, 14, 15).

In the present work, we combine transcriptome-wide RNA sequencing data from 362 corpus and 342 antrum mucosa samples with genome-wide genotyping data to comprehensively characterize genetically regulated gene expression of the proximal and distal stomach. This study is the first that considers eQTLs of both major gastric sites covering oxyntic and antral mucosa types that were derived from healthy individuals. The use of nondisease tissue allows to investigate normal tissue physiology as well as disease etiology by comparing genome-wide association study (GWAS) signals with eQTLs. As samples were collected from regular biopsies, the gene expression analysis is potentially less biased with respect to biological processes that might be activated in postmortem tissues (as such in GTEx data). The aim of our study was to better understand the normal physiological state of the two major gastric sites by investigating tissue-specific gene expression and gene regulation as well as their contribution to pathophysiology. For the latter, we analyzed whether the genetic regulation of the gastric transcriptome is involved in the susceptibility to metabolic phenotypes and we strived to identify candidate genes associated with metabolic traits and diseases by prioritizing GWAS loci through transcriptome analysis of gastric tissues.

## MATERIALS AND METHODS

### Study Design and Sample Collection

Subject recruitment took place at nine sites between 2016 and 2017. Four hundred thirty-one individuals (185 males and 246 females) were included in the study who were all of European ancestry and underwent a gastroscopy due to unclear upper abdominal symptoms. The average age of participants was 52.8 ± 15.1 yr. Apart from diagnostic biopsies, mucosa biopsy specimens from five sites of the stomach (cardia, corpus, fundus, antrum, and angulus) were collected from each individual. Subjects were excluded from the study if histopathologic examination of the gastric mucosa at the Institute of Pathology in Bayreuth, Germany, revealed *Helicobacter pylori* infection. In addition, other medical conditions leading to the unclear upper abdominal symptoms had to be excluded in order to sustain a participant’s study inclusion. This implied a normal complete blood count, among others. Although 128 participants used proton pump inhibitors to ease the upper abdominal symptoms, the long-term use of medication was an exclusion criterium. Detailed sample metadata are available in Supplemental Table S1. All mucosa biopsies were preserved in RNAlater solution (ThermoFisher) and stored at −20°C until nucleic acid extraction. Informed consent was obtained from all participants and a study approval was obtained from the ethic board at the University of Bonn, Germany.

### RNA-Seq Library Preparation, Sequencing, and Data Processing

Pre-experiments showed that the expression in corpus and antrum covers most transcriptomic variance of the five gastric sites (16). Thus, 410 corpus and 381 antrum mucosa samples were subjected to DNA and RNA extraction as well as RNA sequencing and genotyping. Tissue samples were mechanically homogenized with ceramic beads and a Precellys tissue homogenizer (Bertin Instruments, France). Genomic DNA and total RNA were extracted using the Allprep DNA/RNA Mini Kit (Qiagen, Germany). For library preparation, the QuantSeq 3′ mRNA-Seq Library Prep Kit FWD for Illumina (Lexogen, Austria) was used. Subsequent single-end sequencing with 50 bp reads was performed on a HiSeq 2500 v4 (Illumina). FastQC v0.11.7 (17) (http://www.bioinformatics.babraham.ac.uk/projects/fastqc/) was used for quality control. Adapter trimming was performed with bbduk from the BBMap v37.44 (18). Trimmed reads were aligned against human transcriptome with STAR Aligner 2.5.2b (19) using the Genome Reference Consortium human reference 38 assembly (GRCh38/hg38) (20). Gene expression was quantified with Feature Counts v1.5.1 (21) using Ensembl (22) annotation GRCh38.89 as reference.

### Incorporation of GTEx Expression Data

For downstream analyses, RNA sequencing gene read counts from GTEx analysis release v8 were downloaded from GTEx portal (https://gtexportal.org/home/downloads/adult-gtex/bulk_tissue_expression) (8). Expression data from K562 leukemia cell line was excluded from the analysis as the cells are not related to any GTEx participant and do not originate from healthy tissue. Whole blood samples were also removed from the data set since the majority of samples appeared to be outliers in principle component analysis. The customized data set represents 14,481 RNA sequencing assays performed on 53 conditions (50 tissues and three derived cell lines). While single-end sequencing was performed of corpus and antrum samples, GTEx samples were sequenced in a paired-end run. To make reads from both library preparation protocols comparable, raw gene read counts from GTEx were transformed into reads per kilobase [rpk(gene) = number of reads mapped to a gene/gene length × 1,000]. Gene reads from both gastric sites were merged with adjusted gene expression data from GTEx. Genes with fewer than 6 counts per million in less than 20% of the samples were considered not expressed and removed from the data set. Samples with library sizes outside of 3 standard deviations were filtered out. Trimmed mean of M values from reads were computed using edgeR (23) to normalize for different library sizes and expression for each gene was normalized by inverse normal transformation. Principal component analysis showed that corpus and antrum samples cluster closest to GTEx stomach samples compared with other GTEx tissues (Supplemental Fig. S1).

### Differential Gene Expression Analysis and Hierarchical Clustering

Differential gene expression analysis was performed with the R/Bioconductor package limma (24). The mean-variance trend was converted into precision weights by the voom function. Tissue as a covariate was regressed out before linear modeling and empirical Bayes moderation. *P* values were corrected for multiple testing using the Benjamini–Hochberg method. False discovery rate (FDR) < 0.01 and absolute log2 fold change > 1.5 were used as cutoffs to consider a gene as differentially expressed. In addition, another differential gene expression analysis was performed as described above only including participants of those expression data of both corpus and antrum was available using the gastric site, the interaction of the gastric site and sex as well as the interaction of the gastric site and proton pump inhibitor (PPI) intake as covariates for the linear model to investigate the influence of the participant’s sex and medication on the study results. The top 100 most variable expressed genes between the two gastric sites corpus and antrum were identified using the rowVars function of the matrixStats (25) R package on log-transformed counts per million expression values to perform unsupervised hierarchical clustering. Expression data of these genes were visualized with the R package pheatmap (26).

### Generation of Tissue-Specific Gene Sets

Tissue-specific gene sets for corpus and antrum were generated similarly to the approach described by Bryois et al. (27). First, stomach samples from GTEx were removed from the merged and filtered expression data set and all nonprotein-coding genes were discarded. Gene-wise normalization of expression data was performed to adjust for different library preparation protocols. Next, mean expression values for each gene in each tissue were computed and used for calculation of relative gene expression specificity which results from dividing the expression of each gene in each tissue by the total expression of that gene in all tissues. Values for relative gene expression specificity of a tissue range from 0 to 1. 0 implies no expression in the respective tissue, whereas 1 means that a gene is exclusively expressed in the given tissue. The top 15% most specific genes (*n* = 2,749) each were used as tissue-specific gene sets of corpus and antrum (Supplemental Tables S2 and S3) for downstream analyses.

### Enrichment Analysis

Different tools to perform enrichment analysis were utilized. The teEnrichment function from the TissueEnrich (28) R package was applied for enrichment analysis of corpus- and antrum-specific genes based on expression levels. The R package clusterProfiler (29) was used to calculate gene ontology enrichment from biological process terms providing differentially expressed genes as input. Disease Ontology enrichment via DOSE was also studied with clusterProfiler. GENE2FUNC from FUMA (30) webtool (https://fuma.ctglab.nl/) was used to visualize tissue-enrichment of the corpus- and antrum-specific gene sets. Enrichr (31) (https://maayanlab.cloud/Enrichr/) was used for pathway analysis which integrates a variety of databases. We focused on the BioPlanet 2019 (32) and MSigDB Hallmark 2020 (33) libraries. For any conducted enrichment analysis, a Benjamini-Hochberg adjusted *P* value < 0.05 was used as significance threshold.

### Genome-Wide Genotyping, Quality Control, and Imputation

Genotyping was performed with the Infinium Global Screening Array v1.0 (GSA, Illumina, San Diego, CA). For the pre-imputation quality control, samples were excluded if they did not meet the following criteria: SNP call rate > 97%, rate of autosomal heterozygosity: mean ±6 standard deviations (SD), rate of X-chromosomal heterozygous genotypes: <2% for a supposed male individual and > 10% for a supposed female individual. Furthermore, PLINK v1.9 (34) and KING (35) were used to uncover the close relationship of individuals. From each pair of individuals with an estimated identity by descent (IBD) probability > 0.2 or kinship coefficient > 0.0884, the sample with the higher missing genotype rate was removed. Individuals appearing as outliers in multidimensional scaling (MDS) analysis were also discarded. SNPs were excluded if the SNP missing rate was > 10%, the *P* value for Hardy–Weinberg equilibrium (HWE) was < 10^−6^, and the minor allele frequency (MAF) was less than 0.01. Genotype imputation to estimate missing SNPs was performed with Impute2 (36) and the European 1000 Genomes Phase 3 LD panel (37) as reference. In the post-imputation quality control, variants were excluded if the information score was less than 0.8, the genotyping rate was < 95% for best-guessed genotypes at posterior probability of more than 0.9, the *P* value for HWE was < 10^−6^, or the MAF was less than 0.01.

### Power Analysis, eQTL Mapping, and Meta-Analysis With GTEx eQTL Data

eQTL power calculations were carried out with the R package powerEQTL v0.3.4 (38) using the simple linear regression approach. To detect cis-eQTLs with a MAF of 0.1 and small effect sizes (β = 0.2) (39) at a power of 0.8, assuming an FDR of 5% and 93,019,174 tested SNP-gene pairs for the corpus data set and 100,787,044 tested SNPs for the antrum data set, a sample size of *n* = 247 is required. Under the same conditions, a sample size of *n* = 320 allows the reliable detection of cis-eQTLs with a MAF of 0.1 and effect sizes as small as 0.16, and a sample size of *n* = 306 the detection of cis-eQTLs with a MAF of 0.05 and effect sizes of 0.25. Three hundred and sixty-two corpus and 342 antrum samples were included in the eQTL analysis after quality control, which thus are adequate sample sizes for the analysis of common variants with moderate gene expression regulatory effects in cis-regions. For eQTL identification, QTLTools (40) was used on the expression and genotype data according to parameters applied by the Genotype-Tissue Expression (GTEx) consortium (2). The models were adjusted for sex, three genotype-based principal components and a set of PEER-covariates derived from the normalized expression data. Cis-eQTLs were mapped within a window of 1 Mb around the transcription start site (TSS) of a respective gene. SNP-gene associations were regarded as significant with a nominal *P* value below a genome-wide empirical *P* value threshold (Pt) determined for each gene by extrapolation from a Beta distribution fitted to adaptive permutations. For meta-analysis, all GTEx v7 eQTL associations for 48 tissues were downloaded from the GTEx portal (https://gtexportal.org/home/downloads/adult-gtex/qtl) (2). Pairwise correlation of the effect sizes of significant eQTLs in corpus and antrum with GTEx tissues was conducted to investigate the SNP effect variation between tissues.

### LD Score Regression

Stratified linkage disequilibrium score regression (LDSC) (41) was conducted to partition SNP-based heritability of traits and diseases and test its enrichment in antrum- and corpus-specific gene sets. All GWAS summary statistics used for LDSC were obtained from the UK biobank made publicly available by the Neale Lab (www.nealelab.is/uk-biobank) (42). First, the LDSC partitioned heritability approach was used to compute gene set enrichment for selected International Classification of Diseases 10th Revision (ICD-10) diseases (*n* = 319). From the 657 GWAS summary statistics available for ICD-10 diseases, 338 diseases without a gastrointestinal etiology were excluded (Supplemental Table S4). Next, targeted enrichment analysis of the SNP-based heritability of metabolism- and obesity-related traits (*n* = 65) by considering the corpus- and antrum-specific gene sets was performed. This analysis involved GWAS summary statistics from two UK biobank (UKBB) categories. From the category “Physical measure summary” (UKBB Category 17518), the subcategories body composition by impedance (*n* = 30), body size measures (*n* = 6), and bone-densitometry of heel (*n* = 3) were included as well as four further metabolic/obesity-related traits, namely comparative body size at age 10, comparative height size at age 10, birth weight, and age when the period started. All traits regarding “Physical measure summary” of arms and legs are present twice in UKBB summary statistics, referring to left and right. For simplicity, only results for the right arm and leg were considered. From the category “Blood chemistry” (UKBB Category 17518), all blood metabolites except hormones and enzymes (*n* = 22) were utilized. Trait-gene set enrichment was considered significant at a Benjamini–Hochberg adjusted *P* value < 0.05. A list with the GWAS summary statistics used for LDSC can be found in Supplemental Table S5.

### Transcriptome-Wide Association Analysis

TWAS analyses were conducted by using functional summary-based imputation (FUSION) software (43) to identify the genes associated with the corpus- and antrum-specific gene set enrichment for metabolic- and obesity-related traits. Expression prediction models for corpus and antrum were computed using variants ±500 kb of each gene. The tissue-related expression weights were combined with the GWAS summary statistics (Supplemental Table S6) to estimate the association between target traits (significantly enriched diseases and traits from LDSC analysis) and gene expression. The European 1000 Genomes Phase 3 LD panel (37) was used as reference. TWAS associations were considered “transcriptome-wide significant” after Bonferroni-correction of the *P* values for the number of tested genes (*P*_corpus_ = 0.05/3,269 = 1.5 × 10^−5^, *P*_antrum_ = 0.05/4,182 = 1.1 × 10^−5^).

### Validation of the TWAS Results

The built-in interface to the COLOC (44) software in FUSION was used to explore whether a single shared variant is responsible for both GWAS and eQTL signals. All genes with transcriptome-wide significance and within 1 Mb of a respective locus were subjected to Bayesian colocalization analysis. COLOC estimates the posterior probability (PP), i.e., the association of GWAS and eQTL signals. PP4 > 0.8 suggests strong evidence of colocalization. In addition, conditional analysis was performed on the transcriptome-wide significant associations using FUSION. This analysis aimed to assess to what extent the GWAS signal is driven by genetically regulated expression. Furthermore, a gene-based finemapping approach using FOCUS (45) was used to prioritize genes with strong causal evidence in TWAS analysis. GWAS summary statistics, eQTL weights (consistent with the eQTL reference panel used for FUSION), and the European 1000 Genomes Phase 3 LD reference data (37) were used to estimate the posterior inclusion probability (PIP) of each gene being a member of a credible set with 90% probability of containing the causal gene. PIP > 0.5 indicates that the gene is more likely to be causally associated than any other gene in the region of interest and thus considered as putatively causal. Moreover, we searched the resulting associated top eQTL in the GWAS Atlas PheWAS database (https://atlas.ctglab.nl/PheWAS) (46), defining *P* < 1 × 10^−9^ as a statistically significant SNP-phenotype association. The GWAS Atlas PheWAS database is a catalog of 4,756 publicly available GWAS summary statistics with the possibility to query single SNPs or genes to identify associated phenotypes.

## RESULTS

### Transcriptome Profiles Differ Between Corpus and Antrum

We aimed to identify differentially expressed genes in corpus and antrum mucosa of healthy individuals to study physiology in both gastric regions. Overall, we found 2,412 genes that were significantly differentially expressed in corpus and 3,508 in antrum compared with the other gastric site and all GTEx tissues except stomach by using Benjamini–Hochberg adjusted *P* value < 0.01 and absolute log2 fold change > 1.5 as thresholds. The top 10 differentially expressed genes in corpus with the highest positive fold change were *PGA5*, *LIPF*, *CHIA*, *PGC*, *GIF*, *ATP4A*, *ATP4B*, *GHRL*, *GKN1*, and *TFF2*. In antrum *GAST*, *GKN1*, *GKN2*, *MUC5AC*, *TFF1*, *MUCL3*, *TFF2*, *NKX6-3*, *NKX6-2*, and *CTSE* were the top 10 differentially expressed genes according to fold change. There were three PPI-related differentially expressed genes between corpus and antrum, i.e., *GUCA2B*, *ALDOB*, and *CELA3A* (Supplemental Table S7), and none that were influenced by participant’s sex. Unsupervised hierarchical clustering of the top 100 most variable expressed genes revealed homogenous transcriptome profiles in corpus and antrum that differed substantially between both gastric sites (Supplemental Fig. S2). We used TissueEnrich (28) to perform tissue-specific gene enrichment analysis and selected genes with at least fivefold higher expression levels in corpus and antrum in comparison to all other GTEx tissues except stomach as signature genes for the respective gastric region. Consistent with the results from the differential gene expression analysis, the corpus signature genes included *CHIA*, *GHRL*, *PGA5*, *LIPF*, *ATP4A*, and *ATP4B* whereas *MUCL3*, *NKX6-2*, *GAST*, and *TFF1* were pointed out as signature genes for antrum. In addition, we generated site-specific gene sets for subsequent analyses containing protein-coding genes with the highest relative expression compared with other tissues according to an approach implemented by Bryois et al.(27) (see materials and methods for details). Each gene set derived from this method consisted of 2,749 genes with an overlap of 1,555 genes (57%) between corpus and antrum (Supplemental Tables S2 and S3). Both gene sets showed the highest enrichment in GTEx stomach upregulated genes confirming the quality of both gene sets (Supplemental Fig. S3, *A* and *B*).

### Functional Enrichment Analyses Reveal Corpus- and Antrum-Specific Physiology

We performed gene ontology and pathway enrichment analysis to gain insights into the biological role of the differentially expressed and site-specific genes in gastric physiology. The enrichment analysis of biological processes using gene ontology (GO) terms revealed the association of differentially expressed genes in corpus with epithelial cell proliferation (*P*_adj_ = 1.71 × 10^−7^) and its regulation (*P*_adj_ = 4.15 × 10^−7^), digestion (*P*_adj_ = 1.71 × 10^−7^), muscle contraction (*P*_adj_ = 1.73 × 10^−6^), and blood circulation (*P*_adj_ = 4.92 × 10^−6^) (Supplemental Fig. S4*A*). The top five GO terms that were overrepresented in differentially expressed genes in antrum were blood circulation (*P*_adj_ = 5.95 × 10^−13^), regulation of hormone levels (*P*_adj_ = 3.0 × 10^−10^), digestion (*P*_adj_ = 4.2 × 10^−10^), epithelial cell proliferation (*P*_adj_ = 1.0 × 10^−9^), and muscle tissue development (*P*_adj_ = 2.31 × 10^−8^) (Supplemental Fig. S4*B*). Full enrichment results are listed in Supplemental Table S8 for corpus and in Supplemental Table S9 for antrum.

Pathway enrichment analysis was performed according to the BioPlanet 2019 and MSigDB Hallmark 2020 libraries as described in a publication by Kim et al. (47). Many pathways overexpressed in the corpus-specific gene set were related to energy metabolism (*P*_adj_ = 2.19 × 10^−13^) (Supplemental Table S10). In addition, gastric acid secretion (*P*_adj_ = 1.32 × 10^−3^), amino acid (*P*_adj_ = 3.95 × 10^−6^), and lipid metabolism (*P*_adj_ = 7.45 × 10^−6^), and essential cellular processes such as protein secretion (*P*_adj_ = 1.17 × 10^−4^) were also enriched for corpus-specific genes. Pathways overrepresented in the antrum-specific gene set were mainly associated with mitosis (*P*_adj_ = 1.14 × 10^−15^) and cell cycle (*P*_adj_ = 1.24 × 10^−10^) but there was also a significant overrepresentation of protein metabolism (*P*_adj_ = 2.38 × 10^−5^), estrogen signaling (*P*_adj_ = 6.97 × 10^−5^), and posttranslational protein modifications (*P*_adj_ = 4.96 × 10^−9^) (Supplemental Table S11).

In a second approach, we compared our corpus expression data to that from GTEx and found 5,794 differentially expressed genes with an absolute log2 fold change > 1.5 and Benjamini-Hochberg adjusted *P* value < 0.01 (Supplemental Table S12). Pathway enrichment analysis according to the BioPlanet 2019 and MSigDB Hallmark 2020 libraries showed that genes that are upregulated in our corpus data set (*n* = 2,149) can be associated with gastric acid secretion (*P*_adj_ = 1.60 × 10^−3^), energy metabolism (*P*_adj_ = 1.60 × 10^−3^), and mitosis (*P*_adj_ = 0.03), among others (Supplemental Table S13).

### Corpus and Antrum Exhibit Tissue-Specific Expression Quantitative Trait Loci

In our analysis, we focused on cis-eQTLs to evaluate gene expression regulation (within 1 Mb from the gene transcriptional start site) as distant regulatory mechanisms (i.e., trans-eQTLs) are more difficult to identify both due to the comparatively smaller biological effects (i.e., they are characterized by lower effect sizes) and the statistical power (i.e., combinatorial explosion of variants to gene combinations). In total, we identified 4,228 cis-regulatory eQTLs in corpus and 5,705 in antrum using a Benjamini–Hochberg adjusted *P* value < 0.05 as the significance threshold (Supplemental Tables S14 and S15). In the corpus, we mapped 4,201 eQTLs regulating the expression of 4,228 genes (eGenes). In the antrum, we found 5,658 eQTLs affecting the expression of 5,705 eGenes. In total 3,179 eGenes were shared between corpus and antrum (*P*_Fisher’s exact test_ < 2.2 × 10^−16^), thus suggesting a strong co-regulation of gene expression in both gastric regions. However, only 428 top-gene eQTLs (4%) were overlapping between corpus and antrum, indicating also some degree of heterogeneity in the gene regulation. Comparing eGenes with our corpus- and antrum-specific gene sets defined by the approach of Bryois et al.(27) (Supplemental Tables S2 and S3), 1,328 (48%) of the antrum-specific genes were eGenes in the antrum and 1,091 (40%) corpus-specific genes were eGenes in the corpus.

Pathway enrichment analysis revealed that genes whose expression is regulated by eQTLs in corpus were associated with metabolism (*P*_adj_ = 3.51 × 10^−12^), in particular fatty acid (*P*_adj_ = 5.05 × 10^−7^) and protein metabolism (*P*_adj_ = 1.43 × 10^−6^), estrogen signaling (*P*_adj_ = 1.20 × 10^−6^), lysosome (*P*_adj_ = 1.79 × 10^−5^), and oxidative phosphorylation (*P*_adj_ = 9.56 × 10^−6^) (Supplemental Table S16). In antrum, genes whose expression is regulated by eQTLs were associated with metabolism (*P*_adj_ = 2.21 × 10^−12^), specifically protein (*P*_adj_ = 7.72 × 10^−4^), lipid (*P*_adj_ = 7.72 × 10^−4^) and bile acid metabolism (*P*_adj_ = 1.37 × 10^−4^), lysosome (*P*_adj_ = 5.49 × 10^−10^) and peroxisome (*P*_adj_ = 9.45 × 10^−5^), and adipogenesis (*P*_adj_ = 9.70 × 10^−6^) (Supplemental Table S17).

**Figure 1.**
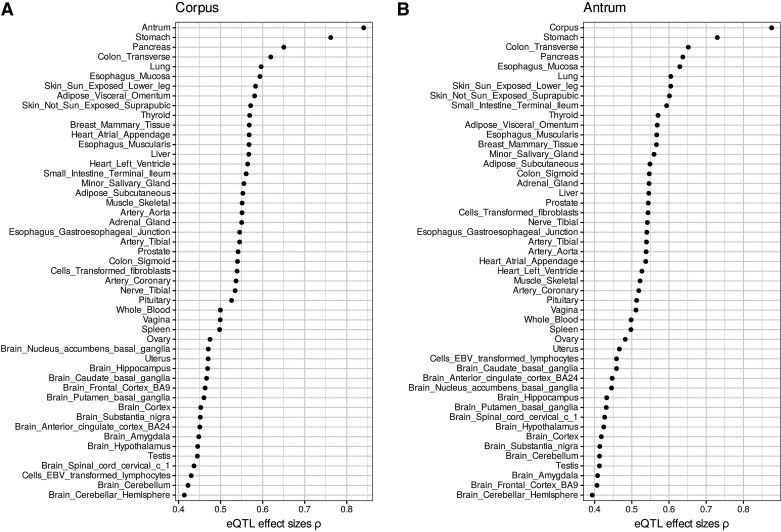
Meta-analysis with the GTEx v7 eQTL data set. Pairwise spearman correlation of eQTL effect sizes between corpus (*A*) or antrum (*B*) and GTEx v7 tissues. We only considered significant variant-gene associations based on permutations and FDR ≤ 0.05. eQTL, expression quantitative trait locus; GTEx, Genotype-Tissue Expression.

It is well known that eQTLs are often shared across different tissues. Meta-analysis with the full GTEx v7 eQTL data set (*n* = 48 tissues) was performed to determine the pairwise correlation of eQTLs between tissues based on their effect sizes. Corpus eQTL effect sizes correlated most to antrum (ρ = 0.84) followed by GTEx stomach (ρ = 0.76), pancreas (ρ = 0.65), and transverse colon (ρ = 0.62) (Fig. 1*A*). The effect sizes of antrum eQTLs showed highest correlation with corpus (ρ = 0.88), GTEx stomach (ρ = 0.73), transverse colon (ρ = 0.65), and pancreas (ρ = 0.64) (Fig. 1*B*). As expected, the correlation of eQTL effect sizes between corpus and GTEx stomach was higher than between antrum and GTEx stomach.

### Disease Ontology Analysis: Enrichment for Cardiovascular and Metabolic Diseases

We performed disease ontology enrichment analysis to evaluate to which extent genes differentially expressed in corpus and antrum are associated with diseases. A total of 28 disease ontology terms were significantly enriched for genes differentially expressed in corpus (Supplemental Table S18) and 130 for differentially expressed genes in antrum (Supplemental Table S19). Apart from the different numbers of disease ontology terms overrepresented in both gastric regions, there was a consistent significant overlap between corpus- and antrum-associated diseases (*P*_Fisher’s exact test_ < 2.2 × 10^−16^). Genes related to cardiovascular diseases like coronary artery disease (*P*_adj,corpus_ = 4.92 × 10^−4^, *P*_adj,antrum_ = 1.23 × 10^−6^), and myocardial infarction (*P*_adj,corpus_ = 2.99 × 10^−4^, *P*_adj,antrum_ = 1.23 × 10^−6^) were enriched in both gastric regions, so were genes associated with metabolic diseases such as overnutrition (*P*_adj,corpus_ = 7.94 × 10^−4^, *P*_adj,antrum_ = 8.3 × 10^−6^), nutrition disease (*P*_adj,corpus_ = 7.94 × 10^−4^, *P*_adj,antrum_ = 1.87 × 10^−6^), and obesity (*P*_adj,corpus_ = 2.67 × 10^−3^, *P*_adj,antrum_ = 2.46 × 10^−5^). *P* values generally seemed to be smaller for antrum.

### Site-Specific Gene Sets: Enrichment for Metabolic, Obesity-Related, and Cardiovascular Diseases and Traits

Following the disease ontology enrichment analysis, we tested whether the genomic regions in which highly and specifically expressed genes are located show enrichment for GWAS signals of metabolic diseases. We, thus, applied stratified linkage disequilibrium score regression (LDSC) to partition the single nucleotide polymorphism (SNP)-based heritability of 319 selected ICD-10 diseases (see materials and methods) across the tissue-specific genes of corpus and antrum.

**Figure 2.**
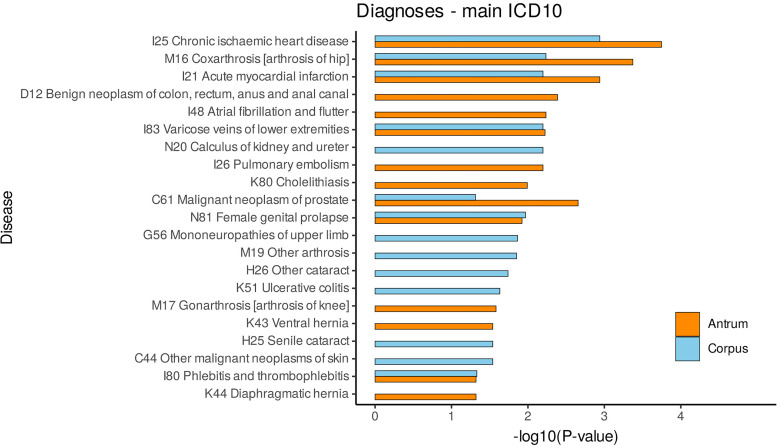
Stratified LDSC results for partitioning SNP-based heritability of selected ICD-10 diseases. Significant enrichments, corrected for multiple testing by the Benjamini–Hochberg method are displayed. −log10(*P*-values) of heritability enrichment for corpus (blue) and antrum (orange). LDSC, linkage disequilibrium score regression; SNP, single nucleotide polymorphism.

We found significant enrichment (Benjamini–Hochberg adjusted *P* value < 0.05) for 14 diseases each in the corpus- and antrum-specific gene sets (Fig. 2). In agreement with our disease ontology enrichment analysis, associated diseases are mostly related to the cardiovascular system, metabolism, and obesity. Again, there was an overlap in disease association of the corpus- and antrum-specific gene sets regarding chronic ischemic heart disease (*P*_adj,corpus_ = 1.15 × 10^−3^, *P*_adj,antrum_ = 1.79 × 10^−4^), coxarthrosis (*P*_adj,corpus_ = 5.82 × 10^−3^, *P*_adj,antrum_ = 4.24 × 10^−4^), acute myocardial infarction (*P*_adj,corpus_ = 6.36 × 10^−3^, *P*_adj,antrum_ = 1.15 × 10^−3^), among others. Corpus-specific genes were also associated with e.g., calculus of kidney and ureter (*P*_adj_ = 6.36 × 10^−3^) and cataract (*P*_adj_ = 0.018), whereas for example atrial fibrillation and flutter (*P*_adj_ = 5.82 × 10^−3^) and pulmonary embolism (*P*_adj_ = 6.36 × 10^−3^) showed enrichment for antrum-specific genes. All LDSC results can be found in Supplemental Table S20.

**Figure 3.**
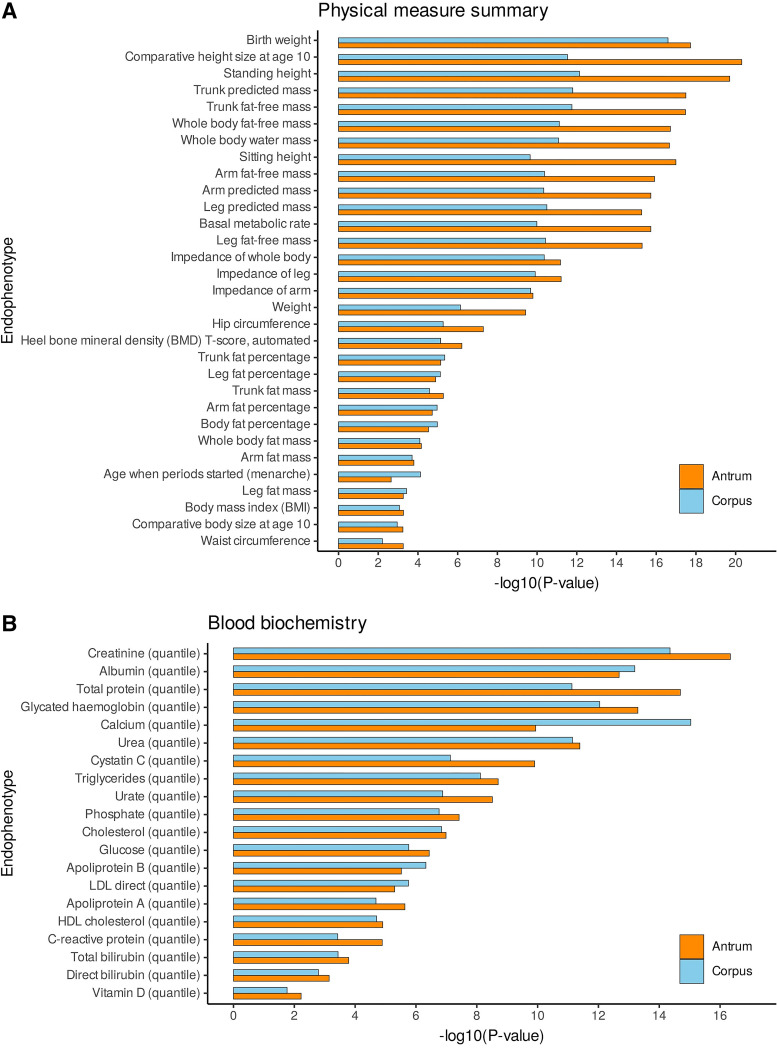
Stratified LDSC results for partitioning SNP-based heritability of metabolism- and obesity-related traits. Significant enrichments of physical measure summary (*A*) and blood biochemistry (*B*), corrected for multiple testing by the Benjamini–Hochberg method are displayed. −log10(*P*-values) of heritability enrichment for corpus (blue) and antrum (orange). LDSC, linkage disequilibrium score regression; SNP, single nucleotide polymorphism.

Next, we aimed to determine endophenotypes potentially being the connection to the disease associations. As cardiovascular and metabolic diseases including obesity are closely linked, we performed targeted enrichment analysis of the SNP-based heritability of 65 selected metabolism- and obesity-related traits belonging to the UK biobank (UKBB) categories physical measure summary and blood chemistry (see materials and methods) by considering the corpus- and antrum-specific gene sets. The significant associations (Benjamini–Hochberg adjusted *P* value < 0.05) of corpus- and antrum-specific genes with selected endophenotypes greatly overlap (*P*_Fisher’s exact test_ = 3.31 × 10^−10^). However, *P* values appeared to be overall smaller for antrum. Significantly associated traits from the category physical measure summary included, among others, weight (*P*_adj,corpus_ = 7.27 × 10^−7^, *P*_adj,antrum_ = 3.81 × x10^−10^), body composition (*P*_adj,corpus_ = 1.6 × 10^−12^, *P*_adj,antrum_ = 3.21 × 10^−18^), as well as hip (*P*_adj,corpus_ = 5.39 × 10^−6^, *P*_adj,antrum_= 5.11 × 10^−8^) and waist circumference (*P*_adj,corpus _= 6.19 × 10^−3^, *P*_adj,antrum_ = 5.50 × 10^−4^) (Fig. 3*A*). Associations regarding blood chemistry were mainly related to protein metabolism, e.g., creatinine (*P*_adj,corpus_ = 4.44 × 10^−15^, *P*_adj,antrum_ = 4.61 × 10^−17^), albumin (*P*_adj,corpus_ = 6.38 × 10^−14^, *P*_adj,antrum_ = 2.09 × 10^−13^), and urea (*P*_adj,corpus_ = 7.16 × 10^−12^, *P*_adj,antrum_ = 4.10 × 10^−12^), or lipid metabolisms such as triglycerides (*P*_adj,corpus_ = 7.65 × 10^−9^, *P*_adj,antrum_ = 1.99 × 10^−9^) and cholesterol (*P*_adj,corpus_ = 1.43 × 10^−7^, *P*_adj,antrum_ = 1.03 × 10^−7^) (Fig. 3*B*). All LDSC results are listed in Supplemental Table S21.

### TWAS: Potential Candidate Genes Affecting Metabolic and Obesity-Related Traits and Diseases

**Figure 4.**
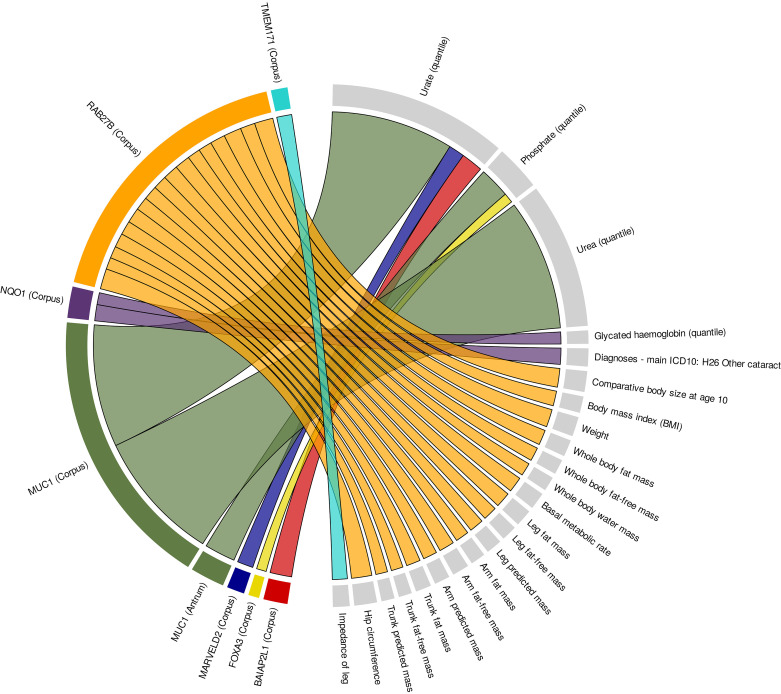
Significant gene-trait/disease associations identified by transcriptome-wide association studies. Ribbons link the genes to the associated metabolic and obesity-related traits and diseases. Each color represents one gene: *BAIAP2L1*: red, *FOXA3*: yellow, *MARVELD2*: blue, *MUC1*: green, *NQO1*: purple, *RAB27B*: orange, *TMEM171*: turquoise. Thickness of the ribbon refers to the −log10(*P*-value) of the associations.

After performing enrichment analysis of highly and specifically expressed genes in corpus and antrum for GWAS signals, we performed a transcriptome-wide association analysis (TWAS) to integrate stomach-specific eQTL effects with GWAS to identify genes whose genetically regulated expression is associated with complex diseases or traits. Our TWAS approach combined expression prediction models for corpus and antrum and GWAS summary statistics of the significantly enriched diseases and traits from the LDSC analysis. In total, we observed 83 significant gene-disease associations using the expression prediction model for corpus and 103 for antrum (Supplemental Table S22). Beyond the level of disease, there were 10,297 gene-trait associations with transcriptome-wide significance using the corpus model and 12,174 using the antrum model (Supplemental Table S23). To ensure the relevant biological function of TWAS hits, further filtering was performed. This included colocalization analysis, finemapping, and conditional analysis which verifies that the associated genes explain most of the GWAS signal at the respective loci (Supplemental Fig. S5). Furthermore, we only included associations where the top1 TWAS model performed best, solely considering the strongest eQTL for each gene to find colocalization of eQTL and GWAS leading variants. Lastly, only stomach-specific genes were considered: *1*) Associated genes had to be part of the tissue-specific gene sets identified by the method of Bryois et al. (27) (Supplemental Tables S2 and S3), *2*) had to have a log2 fold change > 2.5 in the respective stomach site by contrast with other metabolically active tissues (adipose tissue, adrenal gland, blood, colon, liver, muscle, pancreas, pituitary, small intestine, and thyroid), and *3*) stomach had to be among the top 10 tissues regarding median gene expression from GTEx. After applying all filter steps, 26 gene-trait/disease associations remained (Fig. 4 and Supplemental Table S24). There was one significant gene-disease association. On 16q22, rs3790083 was the best eQTL associated with the expression level of *NQO1* in corpus which could be related to cataract risk (*P* = 1.2 × 10^−9^) as well as serum glycated hemoglobin (HbA1c) levels (*P* = 2.68 × 10^−7^). In addition, we found 23 gene-trait associations using the corpus data set and one gene-trait association using the antrum data set. In the corpus gene expression model, we observed the predicted expression of *BAIAP2L1* (*P* = 1.06 × 10^−12^), *MARVELD2* (*P* = 1.71 × 10^−9^), and *MUC1* (*P* = 3.1 × 10^−68^) to be associated with serum urate concentrations. *MUC1* expression was additionally associated with serum urea concentrations (*P* = 4.5 × 10^−73^). Using the antrum gene expression model, genetically predicted expression of *MUC1* was associated with serum phosphate levels (*P* = 1.03 × 10^−17^). Predicted *RAB27B* expression in corpus regulated by rs12456731 showed significant association with hip circumference (*P* = 5.9 × 10^−12^), comparative body size at age 10 (*P* = 5.31 × 10^−11^), weight (*P* = 1.97 × 10^−10^), basal metabolic rate (*P* = 3.53 × 10^−9^), body composition like leg (*P* = 4.18 × 10^−9^) or whole body fat mass (*P* = 5.16 × 10^−9^), and BMI (*P* = 7.17 × 10^−9^). Furthermore, we discovered an association of genetically predicted *FOXA3* expression with serum phosphate concentrations (*P* = 4.03 × 10^−6^), and *TMEM171* expression with leg impedance (*P* = 5.64 × 10^−9^) using the corpus model.

In addition, phenome-wide association studies (PheWAS) were conducted to underpin the TWAS results by identifying associations between the top TWAS eQTLs and phenotypes from a set of curated human phenotypes, the so-called “phenome” (48). The results independently confirmed the association of rs2075571 at *MUC1* with serum urea nitrogen (*P* = 1.94 × 10^−50^) and serum urate concentrations (*P* = 3.78 × 10^−14^) (Supplemental Fig. S6*A*) as well as the association of rs12456731 at *RAB27B* with BMI (*P* = 9.52 × 10^−17^), weight (*P* = 1.02 × 10^−13^), hip circumference (*P* = 6.15 × 10^−13^), comparative body size at age 10 (*P* = 4.34 × 10^−12^), basal metabolic rate (*P* = 5.14 × 10^−12^), and impedance measurements (*P* = 5.76 × 10^−12^) (Supplemental Fig. S6*B*). All PheWAS results for rs2075571 and rs12456731 are available in Supplemental Tables S25 and S26.

Exemplary integrative analysis of GWAS and eQTL data for the most significant phenotypes from PheWAS showed correlation of the *P* values of the variants associated with serum urea levels and *MUC1* eQTLs (R = 0.76, *P* < 2.2 × 10^−16^) (Supplemental Fig. S7*A*) as well as between *RAB27B* eQTLs and BMI-associated variants (*R* = 0.36, *P* < 2.2 × 10^−16^) (Supplemental Fig. S7*B*). In both cases, the identified top eQTLs from TWAS, namely rs2075571 and rs12456731, were both among the top GWAS hits for the given trait and top eQTLs of the respective gene according to *P* value.

**Figure 5.**
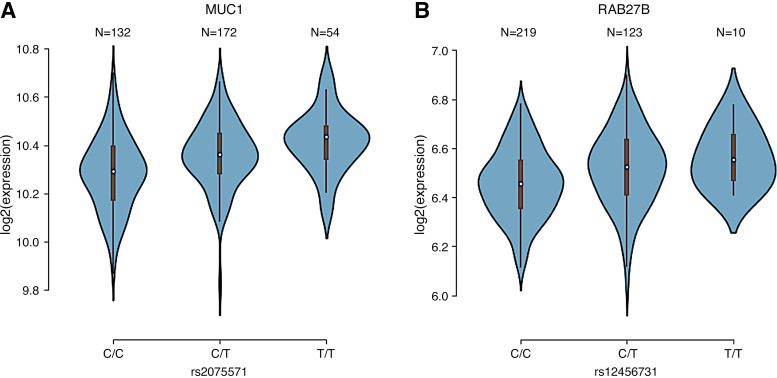
Exemplary eQTL in corpus. Violin plots of associations between genotypes of rs2075571 and *MUC1* expression (*A*) and genotypes of rs12456731 and *RAB27B* expression (*B*). C and T alleles indicate the reference and alternative allele types. The plots indicate the density distribution of the samples in each genotype. The white dot in the box plot (black) shows the median value of the gene expression at each genotype. Number of subjects for each genotype shown above violin. eQTL, expression quantitative trait locus.

Eventually, we could integrate genetics, genomics, and transcriptomics to refine our understanding of the effect of these two loci. The rs2075571 genotype affected the expression of *MUC1* in corpus whereby the presence of the minor allele (T) increased *MUC1* expression (Fig. 5*A*, Supplemental Table S27). The genetically regulated *MUC1* expression in the corpus was shown to be associated with serum urea levels. Expression of *RAB27B* was cis-regulated by the genotype at rs12456731 (Fig. 5*B*, Supplemental Table S27). Carrying the minor allele (T) resulted in higher *RAB27B* expression in the corpus which could be linked to a variety of metabolic traits.

## DISCUSSION

In this study, we provide a large data set comprising genotype and transcriptome data from gastric corpus and for the first time also from gastric antrum. Our findings are, thus, representing both oxyntic and antral mucosa. All tissues were collected from up to 431 healthy individuals which should better facilitate the investigation of tissue physiology and pathology than GTEx samples that were collected post-mortem. This was supported by pathway enrichment analysis of differentially expressed genes between the present and GTEx corpus expression data revealing an enrichment of gastric acid secretion, energy metabolism, and mitosis for genes that are strongly expressed in our corpus data set. All of these are essential for stomach function that will be described in more detailed in the following.

We aimed to clarify the contribution of the different stomach sites to overall organ physiology by identifying corpus- and antrum-specific genes and analyzing gene ontology and pathway enrichment. Corpus and antrum gene expression showed a relatively high heterogeneity as 2,412 genes were differentially expressed in corpus and 3,508 in antrum. However, also a significant enrichment of overlapping expressed genes was observed between both sites, which is expected for related tissues (27). As a consequence, the results from tissue enrichment analysis were partly overlapping. However, also specific pathways for both gastric sites were identified which is particularly interesting for the antrum that has not been analyzed before. Sex and PPI intake do not appear to be confounding factors of the analyses. In fact, despite a sex-specific component is present in gene expression, no sex-specific differences were observed between corpus and antrum. Instead, three genes appeared to be modulated in only one gastric site by PPI intake but none of them was found to be associated with the analyzed phenotypes in our work.

Differentially expressed genes from both stomach sites showed an association with digestion, muscle physiology, blood circulation, and epithelial cell proliferation. The stomach is a hollow muscular organ of the digestive system where food is mixed with acid and enzymes. Gastric blood circulation sustains its normal physiologic structure and function and helps to protect the gastric mucosa against ulcer formation (49). Under physiological conditions, the gastric epithelium is dynamically renewed every 3 to 5 days (50), whereas the turnover kinetics appear to be more rapid in the antrum compared with the corpus (51), which is validated by a strong and specific overrepresentation of cell cycle- and mitosis-related pathways among our antrum-specific genes. Most of the highly corpus-specific genes are related to the gastric juice which is composed of water, hydrochloric acid, digestive enzymes, intrinsic factor, and mucus, meaning that the ingredients of the gastric juice are mainly secreted by glands found in the corpus (52). Gastric acid secretion is a very energy-demanding process and requires the generation of large amounts of adenosine triphosphate (ATP) which is why acid-secreting cells are rich in mitochondria (53). For corpus-specific genes, this explains the enrichment of pathways related to energy metabolism, such as oxidative phosphorylation that is the main source of ATP in eukaryotic cells. Overrepresentation of corpus-specific genes in amino acid and lipid metabolism confirms the secretion of proteases and gastric lipase as components of the gastric juice. In summary, our findings support the assumption that transcriptome profiles differ between corpus and antrum which is due to different tissue physiology. Gastric corpus mucosa contains oxyntic glands that are more involved in digestion, whereas antral mucosa contributes more to epithelial protection and restitution.

The identification of eQTLs helps to understand the genetic regulation of gene expression. Thus, genetic regulatory profiles of corpus and antrum were investigated by cis-eQTL analysis. Although the sample sizes of *n* = 362 for corpus and *n* = 342 for antrum are sufficient to detect common eQTLs with effect sizes as small as 0.16, the power to detect rare eQTLs or eQTLs with very small effect sizes is limited. However, we found that the expression of a large fraction of corpus- and antrum-specific genes is regulated by eQTLs (40% and 48%). This shows that gastric physiology is substantially influenced by genetic variability. The meta-analysis with the GTEx data set showed a strong correlation of corpus and antrum eQTLs with those of GTEx stomach and other organs from the gastrointestinal tract that are rich in mucosa such as transverse colon. This highlights the robustness of our data even though there are some limitations due to differences regarding our and the analysis pipeline used by GTEx. Pathway enrichment analysis demonstrated that genes regulated by cis-eQTLs in corpus were associated with oxidative phosphorylation which is crucial for gastric acid secretion and stomach motility. In contrast, genes involved in lipid and protein metabolism were enriched for both corpus and antrum eQTLs.

Disease ontology enrichment analysis as well as LDSC consistently showed that corpus- and antrum-specific genes were associated with metabolic, obesity-related, and cardiovascular traits and diseases. Using the partitioned heritability approach implemented in LDSC, we were also able to uncover associated endophenotypes by trying to establish the link between the corpus- and antrum-specific genes and associated diseases. We found that the transcriptome profiles in corpus and antrum are correlated with GWAS for many UKBB traits from the category body composition, e.g., weight and body fat distribution, and for many traits from the category blood metabolites, e.g., proteins and lipids. It, thus, seems plausible that these traits promote risk for metabolic, obesity-related, and cardiovascular disease through genetically regulated transcriptome processes in gastric corpus and antrum mucosa.

The final step was the identification of putative candidate genes associated with the traits and diseases outlined above. Although it was not possible to prioritize target genes for all associated traits and diseases, we found some plausible effector genes that we would like to illustrate in the following. Genetically predicted *NQO1* expression in the corpus was shown to be associated with the risk of cataract and serum HbA1c levels. There is evidence that higher HbA1c concentrations increase the risk for the development of cataracts (54). We, thus, hypothesize that allele G of rs3790083 confers risk to cataract via increased serum HbA1c concentrations (Supplemental Tables S24 and S27) and increased *NQO1* expression in the gastric corpus (Supplemental Table S27). Predicted *MUC1* expression in corpus regulated by rs2075571 showed a significant association with serum urate and serum urea levels. Urate is the final product of purine metabolism and has beneficial properties in the human body but an excess of urate is a risk factor for gout (55). Accordingly, rs12411216 and rs4072037 at the *MUC1* locus have been shown to be associated with blood urate levels and gout in several GWAS in the East Asian population (56–58). In East Asians, both variants are in strong linkage disequilibrium (LD) with rs2075571 (*r*^2^ > 0.88) which represents the eQTL for the expression of *MUC1* in gastric corpus. Our data, thus, imply that allele T of rs2075571 leads to decreased serum urate levels via an increased expression of *MUC1* in the corpus mucosa (Supplemental Table S24), which might have protective effects against gout. Apart from serum urate, allele T of rs2075571 was also associated with decreased serum urea levels (Supplemental Tables S24 and S27), which is a product of amino acid metabolism. In a previous GWAS (58), it has been already shown in East Asians that rs12411216 and rs4072037 at *MUC1* are associated with blood urea levels. We, thus, confirm this finding in the European population and show that this effect is most probably due to an eQTL effect on *MUC1* expression in the gastric corpus. Furthermore, we could show that variants for various BMI-related traits and *RAB27B* eQTLs, especially rs12456731, colocalize. This provides evidence that the BMI association signal acts through the regulation of *RAB27B* expression in gastric corpus. In a previous study, rs8092503, which is in strong LD to rs12456731 (*r*^2^ > 0.95), was identified as a risk variant for childhood BMI (59). However, the authors of this GWAS were not able to prioritize a candidate gene at this locus. Our data now suggest that allele T of rs12456731 leads to an increased expression of *RAB27B* in the gastric corpus and thereby increases the risk for different obesity-related traits (Supplemental Tables S24, S26, and S27) and childhood BMI.

In summary, by analyzing transcriptome and genetic regulatory profiles, our findings shed light on distinct and common physiological processes of the corpus and antrum mucosa. Furthermore, our findings demonstrate that the genetic regulation of the gastric corpus and antrum transcriptome is associated with numerous metabolic, obesity-related, and cardiovascular traits and diseases. Our data, thus, provide important insights into the genetic regulation of gastric physiology and pathophysiology. Future studies are now needed to elucidate which pathways and biological mechanisms contribute in detail to the observed outcomes at the cellular level. For this and other scientific questions, the here presented transcriptome data from corpus and antral mucosa is publicly available at Gene Expression Omnibus, accession number GSE242339.

## DATA AVAILABILITY

The gene expression data are publicly available at Gene Expression Omnibus under Accession No. GSE242339.

## SUPPLEMENTAL DATA

10.6084/m9.figshare.24298435.v1 Supplemental Figs. S1–S7: https://doi.org/10.6084/m9.figshare.24298435.v1.

10.6084/m9.figshare.24298096.v1 Supplemental Tables S1–S27: https://doi.org/10.6084/m9.figshare.24298096.v1.

## GRANTS

This work was supported by the German Research Foundation (SCHU 1596/6-1 and VE 917/1-1). Open Access funding was provided by the Open Access Publishing Fund of Philipps-Universität Marburg.

## DISCLOSURES

No conflicts of interest, financial or otherwise, are declared by the authors.

## AUTHOR CONTRIBUTIONS

T.H., M.V., M.V., J.S., and C.M. conceived and designed research; T.H., M.G., F.D., J.H., M.S., and D.P. performed experiments; L.L.K., J.G., and V.S. analyzed data; L.L.K., A.-S.G., J.B., J.S., and C.M. interpreted results of experiments; L.L.K. prepared figures; L.L.K., J.S., and C.M. drafted manuscript; L.L.K., J.S., and C.M. edited and revised manuscript; L.L.K., T.H., A.-S.G., J.B., J.G., V.S., M.G., F.L.D., J.H., M.S., D.P., M.V., M.V., J.S., and C.M. approved final version of manuscript.
